# One Law Only

**Published:** 1887-11

**Authors:** 


### HOMŒOPATHIC PHYSICIAN,

A MONTHLY JOURNAL OF MEDICAL SCIENCE.

"If our school ever gives up the strict inductive method of Hahnemann, we are lost, and deserve only to be mentioned as a caricature in the history of medicine."—CONSTANTINE HERING.

Vol. VII. NOVEMBER, 1887. No. 11.

### EDITORIAL NOTES.

ONE LAW ONLY.—We feel sure our readers will peruse with pleasure the letter of Professor T. F. Allen, published in this issue. It is gratifying to note that even a physician of Dr. Allen's abilities cannot defend eclectic practices. All departures from homœopathic law are, he admits, confessions of failure on the part of the physician. Dr. Allen says he "knows of no other *law of cure*" than the law of similars. Nor do we. The admission is made that "other methods of treating the sick are forced upon me." The argument made to defend these departures from the one *law of cure* is neither new nor strong. It is simply this: Other methods are forced upon the physician whenever he cannot find the proper homœopathic remedy! In some cases this inability to find the simillimum may be due to the insufficiency of the materia medica, but, as the earlier homœopaths, with a scantier materia medica, were able to do great cure-work, we cannot fail to lay the cause of these failures upon the physician rather than the materia medica. Euthanasia can be produced by the homœopathic remedy, and in such cases it gives more relief and greater aid to the dying patient than any mere palliative. Old-school records do not prove that palliation in cancers, etc., is productive of much comfort. In cases of intermittent fever rendered violent by "inherited psora," Quinia proves a poor reliance. There are many persons who have suffered for years and years with such fevers, and Quinia helped them not at all. A young lady who had "chills" for eight years used

Quinia and Arsenic *ad libitum ;* no cure. These cases are legion. What would Dr. Allen do for them? Shall we try to "stop (*i. e.*, suppress) the overpowering activity of the zymosis and then cure the patient"? Can such a thing be done, Dr. Allen? Can you suppress the disease and *then* cure it, save by first reproducing the disease you have suppressed? In short, it may be said that the cases for which Dr. Allen recommends Quinia are the very ones for which it is not suited. Quinia never cures cases complicated by "inherited psora." We are also advised to cure "a fresh case of gonorrhœa * * * in twenty-four to forty-eight hours" by injections of corrosive Mercury. And if a case or so gets orchitis or rheumatism, the water was too cold!! Does cold water cause such sequelæ as chronic rheumatism, cystic and prostatic disease?* Of gonorrhœa Dr. Post writes: "A large proportion of cases require many weeks and months before the discharge ceases;" * * * "prognosis in every case must be guarded. Complications are by no means always absent in cases that commence very mildly." Dr. Otis "uses no injections until the discharge begins to decline." These allopaths, apparently, do not cure their cases in twenty-four hours or so!

Under the head of local applications, cleansing and washing of sores are mentioned. These phrases are too indefinite to be considered. But we may notice that the color, odor, etc., of discharges are of value in prescribing, and should not be changed by local applications. We are careful to note the peculiarities of the expectoration, of the alvine discharges, of a leucorrhœa, etc. Why, then, neglect to do so in cases of sores, ulcers, and such like?

One potent cause of the use of local applications is that these sores, being external, *can* be *so* treated. "Tangible remedies," said Dr. Matthews Duncan, "are the favorites of the physician and the vulgar." If we prescribe for the patient rather than for "an ulcer," we would cure such cases without resort to local applications.

We most heartily agree with Dr. Allen in believing "that as years go by and good, honest work is done by us, we shall have less need of adjuvants and expedients." And we may add, the use of adjuvants and expedients is not "good, honest work." "What we have to do is to demonstrate the truth of God's law in therapeutics, not to cover it up with dust."

---

* Not to mention gonorrhœal affections of heart, peritoneum, and pleura, of the dura mater and sheath of spinal cord, or gonorrhœal pyæmia, pyelitis, etc., which some observers, like Dr. J. L. Milton, of St. John's Hospital, London, claim may follow badly treated cases.

